# Patterns of Vocal Activity of the Chinese Bamboo Partridge Using BirdNET Analyzer

**DOI:** 10.3390/ani16020303

**Published:** 2026-01-19

**Authors:** Jinjuan Mei, Lingna Li, Wenwen Zhang, Jie Shi, Shengjun Zhao, Fan Yong, Xiaomin Ge, Wenjun Tong, Xu Zhou, Peng Cui

**Affiliations:** 1 State Environmental Protection Key Laboratory on Biodiversity and Biosafety, Research Center for Biodiversity Conservation and Biosafety, Nanjing Institute of Environmental Sciences, Ministry of Ecology and Environment, Nanjing 210042, China; may1111@mail.ustc.edu.cn (J.M.); 8241811887@njfu.edu.cn (L.L.); zhangww1023@126.com (W.Z.); shijie.ahu@gmail.com (J.S.); zhaoshengjun1216@126.com (S.Z.); ychenf@163.com (F.Y.); gexiaomin18@163.com (X.G.); twj@nies.org (W.T.); zhouxu_1359@163.com (X.Z.); 2 The Co-Innovation Center for Sustainable Forestry in Southern China, College of Life Sciences, Nanjing Forestry University, Nanjing 210037, China; 3 Zhejiang Oujiangyuan State Integrated Monitoring Station for Ecological Quality of Forest Ecosystem, Lishui 323000, China; 4 Fujian Wuyishan State Integrated Monitoring Station for Ecological Quality of Forest Ecosystem, Wuyishan 354300, China

**Keywords:** diurnal and seasonal patterns, passive acoustic monitoring, BirdNET, random forest, Xiaohuangshan (XHS)

## Abstract

Passive acoustic monitoring (PAM) is an automatic and non-invasive method for long-term monitoring of bird vocal activity. PAM generates a large amount of data, and the automatic recognition of data poses significant challenges. BirdNET is a free-to-use sound algorithm. We evaluated the effectiveness of BirdNET in identifying the vocalizations of Chinese Bamboo Partridge (a Chinese endemic species) and proposed a random forest (RF) method to improve the result based on the detection of BirdNET. The diurnal and seasonal patterns of calling activity were described based on the identification results. The results showed that the recall of BirdNET-Analyzer was 16.6%, the precision of BirdNET-Analyzer-XHS was 50.8%, and the recall and precision of the RF model were 75.2% and 74.4%, respectively. The diurnal vocal activity of the Chinese Bamboo Partridge showed a bimodal pattern, with peaks around sunrise and sunset and low vocal activity during the central hours of the day. The seasonal vocal activity displayed a unimodal pattern, with a peak in vocal activity during April and May. This study used the Chinese Bamboo Partridge as an example and proposes an improved RF model, built on BirdNET recognition results, for species identification, providing a practical approach for recognizing the vocalizations of regional species.

## Simple Summary

Passive acoustic monitoring (PAM) detects bird vocal activities by collecting acoustic data, which is an automatic and non-invasive method for long-term monitoring. PAM generates a significant amount of data, and the automatic recognition of data poses challenges. BirdNET is a free-to-use sound algorithm to identify acoustic data automatically. In this study, we established the BirdNET model based on the local species acoustic data (BirdNET-Analyzer-XHS). Then we established a random forest (RF) classification model based on acoustic features. Finally, we evaluated the ability of BirdNET and RF models to identify the vocalizations of Chinese Bamboo Partridge (*Bambusicola thoracicus*). The results showed that the recall rate of BirdNET-Analyzer was 16.6%, the precision of BirdNET-Analyzer-XHS was 50.8%, and the recall rate and precision of the RF model were 75.2% and 74.4%, respectively. This study provided a practical method for recognizing the vocalizations of regional species. Based on the identification result, the diurnal and seasonal patterns of vocal activity during the breeding season were analyzed. These findings provide a foundation for applying automatic sound analysis technology to dynamic monitoring and behavioral research on pheasants.

## 1. Introduction

Vocal activity of birds plays an important role in attracting mates and defending territories [1,2]. The seasonal pattern of bird vocalization activity is closely associated with its primary function. When vocalizations serve to attract mates, a peak in vocal activity typically occurs during the early stages of the breeding season. However, if the function of vocal signals is to defend territories, then the vocalization activity remains constant throughout all the seasons [3].

The daily pattern of bird vocal activities usually shows a bimodal pattern, with peaks during the dawn and dusk chorus. There are different hypotheses for dawn chorus: the environmental conditions during dawn time (lower temperature, less air turbulence and lower light) are favorable for sound transmission [4] and not good for foraging; dawn chorus is optimal to defending territories and attracting mates [2,5]; and the song rate can inflect based on the quality and condition of males, since singing earlier at dawn than average is costly, and the dawn chorus may promote a handicap mechanism that prevents dishonest signaling [2,6]. Monitoring the distribution patterns of bird vocalization can contribute to the study of bird behavioral ecology and ecological conservation.

Passive acoustic monitoring provides a useful method for long-term monitoring of bird vocalizations [7,8]. It involves installing automated acoustic recorders in the field to collect audio data, which are then analyzed to determine the sources of sound, their spatial and temporal distribution, and the interactions between the biological activity and environmental factors [9]. Passive acoustic monitoring has been widely applied in different ecosystems (such as marine and forest ecosystems) [7,10] and different biological groups (such as birds, anurans, fishes, etc.) [11,12]. In bird-related studies, passive acoustic monitoring is widely used to detect rare or cryptic species activities, monitor bird diversity, study bird behavior and its relationship with the environment, and many other aspects [13,14,15,16].

Compared with traditional point count recording methods, passive acoustic monitoring has advantages: it is non-invasive, inexpensive, and suitable for long-term monitoring; the data can be standardized and archived; and it has high spatiotemporal efficiency [13,17,18,19]. However, passive acoustic monitoring generates large amounts of audio data, posing challenges for data processing technology [18]. The development in passive acoustic monitoring relies on automatic signal detection software [20,21,22,23], and some studies have improved techniques and analytical methods based on automatic acoustic detection in birds [24]. Among the numerous automatic audio signal detection tools, the free machine learning tool BirdNET is particularly worthy of note [25].

BirdNET employs deep neural network algorithms for the automated detection and classification of over 6500 wildlife species [22,25]. Compared with other automatic detection software, a key advantage of BirdNET is that its recognizer is readily available and can be easily operated through the graphical interface on the Windows platform. It is also user-friendly for people who are not familiar with deep learning principles and have no programming foundation. However, our current understanding of BirdNET’s performance on actual monitoring data is still limited, and it needs further validation [25].

In this study, we evaluated the effectiveness of BirdNET in identifying the vocalizations of Chinese Bamboo Partridge (*Bambusicola thoracicus*) and proposed a random forest-based method to improve the results obtained from BirdNET detections. The Chinese Bamboo Partridge is a Chinese endemic species [26], and it has fewer recordings available in public sound libraries, as these circumstances may decrease the precision or recall of BirdNET. The objective of this study was to provide a practical approach for recognizing the vocalizations of regional species. This study also aimed to investigate the diurnal and seasonal patterns of vocal activity of the Chinese Bamboo Partridge during the breeding season based on the identification result obtained from BirdNET and the random forest model (RF).

## 2. Materials and Methods

### 2.1. Study Area and Study Species

Xiaohuangshan is located in the northwest of Xinbei District, Changzhou City (32°02′ N, 119°50′ E (the location can be found in Figure S1), with the highest elevation of 95 m, close to the Yangtze River, and a total area of about 4.5 km^2^. The region has a subtropical monsoon climate, characterized by hot summers, cold winters, distinct four seasons, and a prominent monsoon. The annual average temperature is 16.2 °C, and the annual average precipitation is 1185.2 mm.

The vegetation type is mainly cultivated deciduous broad-leaved forests. Habitat types include forests, shrubs, grasslands, farmland, ponds, etc. The historical biodiversity survey report shows that there are 537 species of higher plants, 10 species of mammals, and 102 species of birds in the region. Pheasants include the Common Pheasant (*Phasianus colchicus*) and the Chinese Bamboo Partridge.

The Chinese Bamboo Partridge is a Chinese endemic species; it belongs to Galliformes, Phasianidae [26]. It has a gray-blue brow and chest, orange cheeks and breast band, and large black spots on brownish-yellow flanks. It is not particularly shy; singing males can be located by their far-carrying song, a rising series of piercing single and triple notes (https://ebird.org/species/chbpar3, accessed on 8 December 2025). Its distribution is limited within the vast areas south of Shaanxi and east of the Sichuan Basin [27]. It is mainly distributed in hilly and mountainous areas, and active in habitats such as broad-leaved forests, mixed forests, coniferous forests, bamboo forests, shrubs, and farmland in mountains. In March each year, the species shifts from concentrated to dispersed activity and begins to establish territories. The peak egg-laying period is from April to May. During the breeding season, females usually lay 6 to 9 eggs, with an incubation period of 16 to 18 days, sometimes extending up to 20 days [28].

According to a study on the characteristics of the song of the Chinese Bamboo Partridge in early spring in Hangzhou, the song of this species generally consists of 5–11 notes, and every note consists of 3 syllables [29]. The sound sounded like “xu ju qu”. At the beginning of the song, the duration of each note was short, and it gradually became longer over time due to the increasing intervals between syllables. The range of frequency was about 1055–3120 Hz [29].

**Figure 1:**
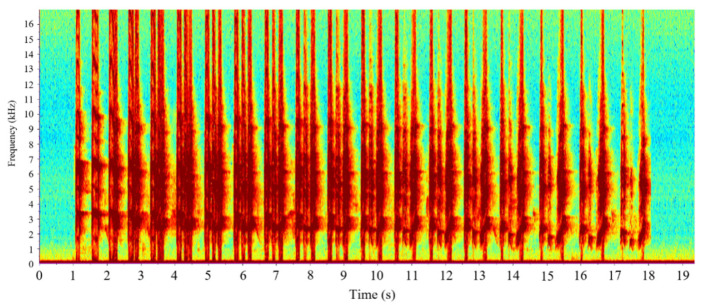
Type 1 vocalizations of the Chinese Bamboo Partridge (*Bambusicola thoracicus*).In the Spectrogram, the X-axis represents the time in seconds, and the Y-axis represents the frequency in kHz. The colors indicate the amplitude or power of corresponding time and frequency.

**Figure 2:**
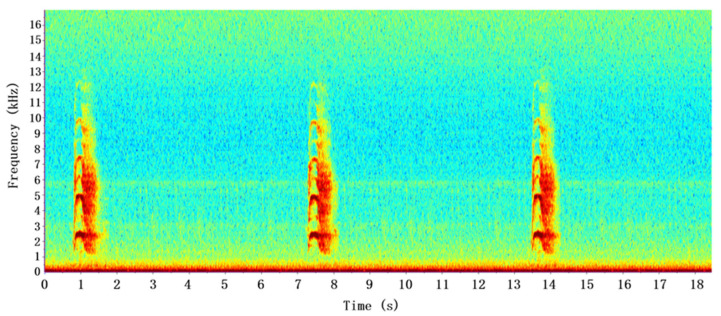
Type 2 vocalizations of the Chinese Bamboo Partridge (*Bambusicola thoracicus*).In the Spectrogram, the X-axis represents the time in seconds, and the Y-axis represents the frequency in kHz. The colors indicate the amplitude or power of corresponding time and frequency.

However, in our study area, the first type of vocalizations of this species contains a larger number of notes (about 16–24) and the duration of the song is longer (about 17–25 s). The sound of the Chinese Bamboo Partridge also exhibits harmonics, but the fundamental frequency remains within this range (1300–4000 Hz). The second type of vocalization of this species sounds like “zhi ya...” and it lasts for a short time. The spectrogram of two types of vocalizations of the Chinese Bamboo Partridge can be found in Figure 1 and Figure 2.

### 2.2. Acoustic Data Collection

We established 3 acoustic monitoring stations that displayed higher vocal activity of the Chinese Bamboo Partridge. The acoustic monitoring stations were located in Xiaohuangshan (Figure S1), and the sound signals were recorded using TN-Bt1 acoustic monitoring equipment (Tianning company, Nanjing, China). The recorder is waterproof (IP68 rating) and has a single internal omnidirectional microphone with a sensitivity of −26 dB. The signal-to-noise ratio (SNR) of the microphone is above 64 dB. It can set four schedules, with start time, end time, recording duration, sleep duration, and sampling rate. The highest sampling rate is 96 kHz. The diameter of the recorders can collect sound effectively within 50 m. The recording files were saved as WAV format in a Micro SD card; the biggest SD card storage capacity is 512 GB. With 15 new AA batteries, it can operate for over 300 h. The operating temperature is between −20 °C and 70 °C.

At each station, the monitoring equipment was fixed on the tree trunk about one meter above the ground. To avoid recording duplicated sounds, the distance between sampling points was more than 1 km. All the recorders were set to record the sounds from 5:00 to 10:00 and 15:00 to 20:00, with a recording cycle of 1 min. Each cycle records for 55 s and sleeps for 5 s. Recordings were conducted continuously from 1 March to 25 June 2024. The recording parameters were set to a sampling rate of 48 kHz and 16 bits per sample, saved in WAV format, and stored on an SD card. Some data were lost due to machine malfunction or depleted batteries. On days when batteries and SD cards were changed, the recordings were incomplete, so only days with complete data were retained in this study. In total, 196 days (approximately 1960 h) of acoustic data were collected. The distribution of these days across different half-month periods can be found in Table S1. The data in each month were divided into three parts; for example, the data from 1 March to 10 March were marked as 3-1 season, and the data from 11 March to 20 March were marked as 3-2 season.

### 2.3. Acoustic Data Analyses

#### 2.3.1. Establishment of Dataset 1

Raven Pro 1.6 software was used to listen to the bird sounds and visualize spectrograms using a Hann window, FFT = 512, overlap 50%, and frame size 100% [30]. We selected the vocalizations of bird species and labeled a species tag to each selection box. All the species tags were checked by the expert observers who are familiar with local bird species. Four characteristics of the bird sounds (begin time, end time, minimum frequency, and maximum frequency) were extracted through the Raven Pro 1.6 software. Finally, a dataset containing 40,270 clips of acoustic data was established, comprising 113 folders (112 bird species and other sounds like background noise and insect sounds). The folder for the Chinese Bamboo Partridge contained 1419 sound clips. We call it dataset 1 in this study.

#### 2.3.2. Establishment of BirdNET-Analyzer-XHS Classification Model

We trained a BirdNET classification model based on dataset 1. The classification model was built via the “Train” tab in the GUI interface of BirdNET (version 2.4, [22]; https://github.com/kahst/BirdNET-Analyzer, accessed on 13 January 2026). We selected training data as dataset 1 (113 classes) and chose Raven as the model output format. BirdNET was run with the following (default) parameters: epoch number = 50, batch size = 32, learning rate = 0.0001. We call this classification model BirdNET-Analyzer-XHS.

#### 2.3.3. Establishment of Dataset 2

We chose 4 days of acoustic data randomly and used two models, BirdNET-Analyzer (BirdNET_GLOBAL_6K_V2.4_Model_Raven) and BirdNET-Analyzer-XHS, to analyze the data. Based on the classification result, we manually corrected and selected the result of the Chinese Bamboo Partridge in Raven. False positives were reassigned to the “other” tag, while false negatives (instances of species’ sounds that were not detected) were relabeled as the Chinese Bamboo Partridge. Then the frequency range was adjusted to 1300–4000 Hz. The sounds of the Chinese Bamboo Partridge have harmonics, but the fundamental frequency lies within this range. Lastly, five characteristics (Freq 25% (Hz), Freq 75% (Hz), Center Freq (Hz), Peak Freq (Hz), and SNR NIST Quick (dB)) were extracted through the Raven Pro 1.6 software. Dataset 2 contained 2536 clips, including 1303 Chinese Bamboo Partridge sound clips and 1233 other sound clips.

#### 2.3.4. Establishment of Random Forest (RF) Classification Model

We trained a random forest (RF) classification model based on dataset 2. Five characteristics were used as predictor variables, and the tag (Chinese Bamboo Partridge or other sound) served as the response vector. Dataset 2 was divided into a training set and a test set at a ratio of 2:1. The RF model was established in R language (V. 4.5.1) [31]. The “randomForest” function in the randomForest package (V 3.1) [32] was used to train the model, while the test set was used to evaluate the performance and decide the proper parameters. In the best fitting model, the number of variables randomly sampled as candidates at each split was 4 (mtry = 4), and the number of trees to grow was 1000 (ntree = 1000). The random forest model was referred to as the RF model in this study.

#### 2.3.5. Establishment of Dataset 3

We randomly chose acoustic data from 3 days and first analyzed it using the BirdNET-Analyzer-XHS model. Then, we used the RF model to analyze the predicted result. Finally, all the results were manually checked (Dataset 3), and the model performance was assessed. Dataset 3 contained 1464 sound clips, including 881 Chinese Bamboo Partridge sound clips and 583 other sound clips.

The sound data in the three datasets are not duplicated.

### 2.4. Assessment of Model Performance

We used recall and precision to assess the model Performance. Recall measures the proportion of actual positive samples that are correctly identified by a model. Recall is calculated using the following formula:

Recall = True Positives/(True Positives + False Negatives)

where True Positives (TPs): The number of positive samples correctly predicted as positive. False negatives (FNs): The number of positive samples incorrectly predicted as negative. A high recall indicates that the model misses very few positive samples.

Precision measures the proportion of predicted positive samples that are actually correct. It is calculated using the following formula:

Precision = True Positives/(True Positives + False Positives)

where True Positives (TPs): The number of positive cases correctly predicted as positive. False positives (FPs): The number of negative cases incorrectly predicted as positive. A high precision indicates that when the model predicts a positive, you can trust that it is likely correct.

Dataset 2 was used to test the performance of BirdNET-Analyzer and BirdNET-Analyzer-XHS. Dataset 3 was used to test the performance of BirdNET-Analyzer-XHS and the RF model.

When assessing the performance of BirdNET, we used default values for the parameters. Minimum Confidence: 0.1; Sensitivity: 1; Overlap: 0. In the BirdNET-Analyzer model, the recall rate was low, so we set a low confidence level (0.1). In contrast, in the BirdNET-Analyzer-XHS model, the precision of the results was low, so a very high confidence score threshold (0.99) was set.

### 2.5. Statistical Analyses

The RF model predictions were used to describe the diurnal and seasonal patterns of vocal activity of the Chinese Bamboo Partridge. The seasonal pattern of vocal activity was described by pooling the data from the three acoustic monitoring stations and was expressed as a percentage of the average number of vocalizations detected per day during different seasons. When studying the daily pattern, the packages “solartime (v 0.0.4)” and “lubridate (v 1.9.4)” in R language (V. 4. 5. 1) [31] were used to change the time to solar time, then the average number of vocalizations per minute in different hours were caculated.

## 3. Results

### 3.1. Prediction Model Performance

The Chinese Bamboo Partridge in dataset 2 was detected by the BirdNET-Analyzer model in 216 out of the 1303 samples with confirmed presence (recall, 16.6%; Table 1); however, there were 3 false positive (FP) recordings with a BirdNET-predicted presence that were not confirmed after verification of the spectrograms (i.e., mislabeled recordings) (precision, 98.6%; Table 1).

The Chinese Bamboo Partridge in dataset 2 was detected by the BirdNET-Analyzer-XHS model in 1271 out of the 1303 samples with a confirmed presence (recall, 97.5%; Table 1), but there were 1233 FP recordings with a BirdNET-Analyzer-XHS-predicted presence that were not confirmed after verification of the spectrograms (mislabeled recordings) (precision, 50.8%; Table 1).

The Chinese Bamboo Partridge in dataset 3 was detected by the BirdNET-Analyzer-XHS model in 851 out of the 881 samples with a confirmed presence (recall, 96.6%; Table 2), but there were 583 FP recordings with a BirdNET-Analyzer-XHS-predicted presence that were not confirmed after verification of the spectrograms (mislabeled recordings) (precision, 59.3%; Table 2).

The Chinese Bamboo Partridge in dataset 3 was detected by the RF model in 640 out of the 851 samples with a confirmed presence (recall, 75.2%; Table 2), although there were 220 FP recordings with an RF predicted presence that were not confirmed after verification of the spectrograms (mislabeled recordings) (precision, 74.4%; Table 2).

In our approach, we first used the BirdNET-Analyzer-XHS model to process the data, followed by the RF model to predict the positive predictions of the first step. Finally, 640 of 881 samples with a confirmed presence were correctly recalled (72.6%), whereas 220 clips were mislabeled (precision, 74.4%).

### 3.2. Diurnal and Seasonal Pattern

#### 3.2.1. Diurnal Pattern

**Figure 3:**
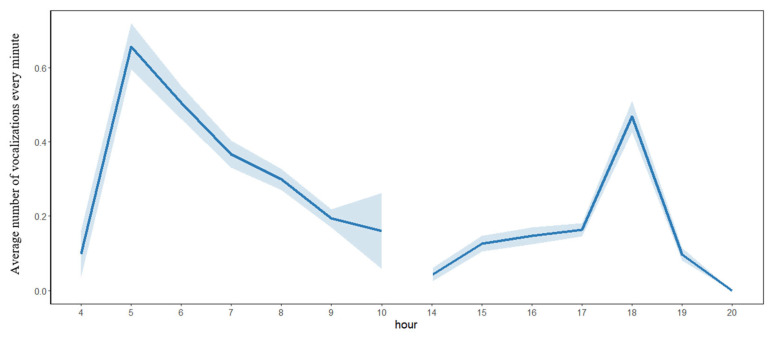
Diurnal pattern of vocal activity of the Chinese Bamboo Partridge (*Bambusicola thoracicus*) in a forest in eastern China. The diurnal patterns of vocal activity are expressed as the average number of sound segments detected per minute in different hours (mean ± 0.95 confidence interval). Vocal activity was monitored via autonomous recording units from 1 March 2024 to 25 June 2024 at three acoustic monitoring stations. The percentage of vocalizations detected per hour can be found in Table S2.

The vocal activity of the Chinese Bamboo Partridge showed a bimodal pattern, with peaks around sunrise and sunset and low vocal activity during the central hours of the day. (Figure 3; for detailed tables of the hourly vocal activity, see Table S2). It showed a higher vocal activity around sunrise, with 38.0% of the vocalizations detected between 4:00 and 7:00, whereas 19.0% of the vocalizations were detected between 17:00 and 19:00 around sunset (Figure 3 and Table S2).

Although it showed bimodal patterns, there are obvious differences in the diurnal pattern of vocal activities of the Chinese Bamboo Partridge in different seasons. At the start time of breeding season (March and the first period of April), it showed a much greater vocal activity around sunset (30.5–38.0% of the vocalizations detected between 18:00 and 19:00 versus 15.1–20.1% of the vocalizations detected between 5:00 and 6:00; Figure 4 and Table S3). In mid-breeding season (second and third period of April, and first and second period of May), the total level of vocal activity became higher and it showed a much greater vocal activity around sunrise (18.6–25.9% of the vocalizations detected between 5:00 and 6:00 versus 3.8–16.9% of the vocalizations detected between 18:00 and 19:00; Figure 4 and Table S3). In the late period of the breeding season (third period of May and June), the total level of vocal activity became lower, and the diurnal pattern changed into a unimodal mode. The peak of vocal activity appeared in the morning (8.6–21.5% of the vocalizations detected between 6:00 and 7:00; Figure 4 and Table S3).

#### 3.2.2. Seasonal Pattern

**Figure 5:**
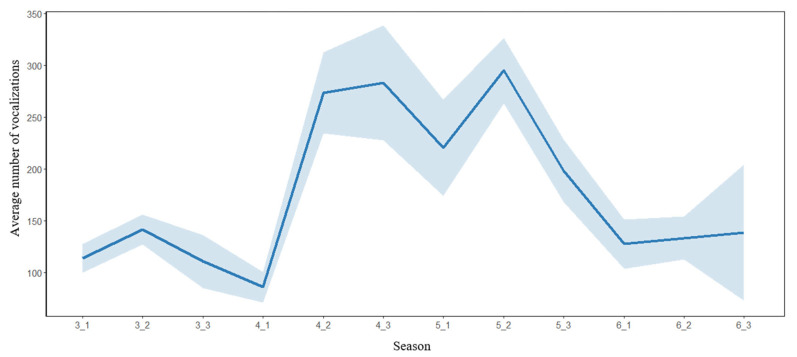
Seasonal pattern of vocal activity of the Chinese Bamboo Partridge in a forest in eastern China. The seasonal pattern of vocal activity was expressed as the percentage of mean vocalizations relative to the total number of vocalizations detected per ten days (mean ± 0.95 confidence interval). The total number and percentage of vocalizations detected per ten days at each station can be found in Table S4. The data in each month were divided into three parts; for example, the data from 1 March to 10 March were marked as 3_1 season, and the data from 11 March to 20 March were marked as 3_2 season.

The vocal activity of the Chinese Bamboo Partridge showed a peak vocal activity occurring between April and May during the mid-breeding season (Figure 5 and Table S4). The percentages of vocalizations detected between the second and third period of April, and first and second period of May, relative to the total vocalizations, were 50.5%.

## 4. Discussion

In this study, we examined the recall and precision of BirdNET-Analyzer and BirdNET-Analyzer-XHS (a BirdNET model established based on local acoustic data) for identifying the vocalizations of the Chinese Bamboo Partridge. BirdNET-Analyzer showed a low recall rate (16.6%), while BirdNET-Analyzer-XHS exhibited only moderate precision (50.8%). The confidence score threshold can affect the recognition results [33,34]. A higher confidence score threshold reduces the recall rate (more false negatives), while a lower threshold reduces the precision (more false positives) [20,35]. In this study, we have set an extremely low confidence level (0.10) in the BirdNET-Analyzer model, but the recall rate remained low. In contrast, a very high confidence score threshold (0.99) was set in the BirdNET-Analyzer-XHS model, but the precision of the results was still low. Therefore, the main factor limiting the accuracy of Chinese Bamboo Partridge sound identification in this study may not be the confidence score threshold.

On the basis of the BirdNET-Analyzer-XHS classification result, we extracted five acoustic features within the fundamental frequency range of the Chinese Bamboo Partridge and established a random forest model. The combination of the two models achieved a recall rate of 72.6% and a precision rate of 74.4%. BirdNET is a large classifier; it is typically optimized for high precision to minimize the false positives, but the recall is likely insufficient to achieve accurate results for some applications that are more sensitive to false negatives, like behavioral research and density estimation [25]. The study solved the problem by building a regional classifier, HawkEars, which is a regional avian classifier for Canada that includes 314 bird and 13 amphibian species, and it had a recall of nearly four times that of BirdNET [36]. Also in this study, the researchers included a separate sub-model for Ruffed Grouse because the recall for this species was near zero in earlier models due to the extremely low frequency of this species’ acoustic signal (∼50 Hz) [36]. In our study, we achieved improved detection accuracy by using BirdNET detection results as input and retraining a random forest classifier on a local dataset.

Although the two-layer model exponentially increases the risk of misjudgment, in prediction results with extremely low false negatives and extremely high false positives, the random forest model can effectively reduce false positives. In the random forest model, “SNR NIST Quick (dB)” is the most important predictor variable, which indirectly indicates that factors such as recording distance and singing direction [33,37], and the background noise level, can have a significant impact on the accuracy of recognition results. In addition, recording distance has been shown to influence the confidence score of BirdNET classification results [33,34].

The type 1 vocalizations of the Chinese Bamboo Partridge are relatively long, lasting about 17–25 s. In this study, the recall rate was calculated using a 3 s timestamp as the minimum unit. However, if a longer time recording is used as the prediction unit to assess whether the model correctly predicted the existence of a species, it may result in higher recall rates and false positives. For example, in an earlier study conducted in a neotropical wetland [35], BirdNET was used to identify the presence of the target species within a 15 min record in files, resulting in a very high recall rate (82.4–92.3%). In this example, if just one clip is correctly predicted, the entire result is considered correct; in contrast, if just one clip incorrectly predicts the presence of a species that does not actually exist, the entire file is mislabeled.

In this study, the precision based on the RF model reached 74.4%, lagging behind the overall precision (79.0%) reported for 984 European and North American bird species via focal recordings [21]. Species with distinctive, relatively simple, and relatively unvaried vocalizations, and more vocalizations in recordings, are easier to identify. The Chinese Bamboo Partridge has two types of vocalizations: type 1 vocalizations have a larger sample size, longer duration, and more distinctive features, so most of the vocalizations identified by the BirdNET model are songs, while the other type is more likely to be missed (false negatives). Although the number of training samples is not the most important predictor of recognition performance [22], there are correlations between the number of training samples and recognizer performance for individual species [38]. Performance has been shown to significantly improve with the iterative addition of only ∼100 samples/species [39]. In future research, the modeling database should include a larger and richer number of vocalizations for each species.

The Chinese Bamboo Partridge showed a bimodal vocal activity pattern, in line with the diurnal patterns described for most bird species (reviewed by [2]), including other sympatric pheasants like Ring-necked Pheasant [40]. Both species displayed the highest peak in vocal activity around dawn and a second, lower peak around dusk. Our results are consistent with earlier descriptions of diurnal activity rhythm based on infrared camera monitoring [41], indicating a primary peak in vocal activity between 05:00 and 06:00 and a secondary peak between 18:00 and 19:00 [42,43].

The intensity of bird vocalizations is related to the reproductive stage. A previous study indicated that after pairs were formed, the activity of male vocalizations was significantly decreased during the fertile period of their females [44], suggesting that bird vocalizations play an important role in attracting mates. Our results indicate that the vocalization activity of the Chinese Bamboo Partridge reached its peak in May. Studies on the breeding behavior of captive Bamboo Partridge showed that the behaviors of courtship and copulation also peak in May, which coincides with the peak period of egg laying [45], indicating that the vocalization of Chinese Bamboo Partridge plays an important role in attracting mates.

Previous studies have shown that the vocalizations of pheasant species play an important role in defending territory and maintaining mate relationships [46]. Our results showed that there are obvious differences in the diurnal pattern of vocal activities of the Chinese Bamboo Partridge in different seasons. At the start time of the breeding season (March and the first half of April), it showed a much greater vocal activity around sunset. At this time, the species shifts from concentrated to dispersed activity and begins to establish territories, and we can infer that at this stage, the main purpose of vocal activity is defending territory. In mid-breeding season (the second half of April and May), the total level of vocal activity becomes higher, and it shows a much greater vocal activity around sunrise. It can be inferred that during the peak period of reproductive behavior, high-intensity vocalizations at sunrise are more inclined to attract mates. The total high level of vocal activity can defend territory at the same time in the second stage. In the late period of the breeding season (June), the total level of vocal activity becomes lower, and the diurnal pattern changes into a unimodal mode, with the peak appearing in the morning. At this stage, the chick brooding work may have been completed; it is more important to save energy and keep safe.

Studies in other Phasianidae species based on infrared camera monitoring also showed similar results: the daily activity patterns of sympatric Elliot’s pheasant (*Syrmaticus ellioti*), silver pheasant (*Lophura nycthemera*), and koklass pheasant (*Pucrasia macrolopha*) in the Qingliangfeng National Nature Reserve, Zhejiang Province, are all different between seasons [38]. Temperature, food supply, and breeding stage can also influence the daily activity patterns [38]. More specific behavioral experiments are needed to determine the different functions of vocalizations at different periods.

## 5. Conclusions

Both the original BirdNET-Analyzer and its regional version, BirdNET-Analyzer-XHS, showed limitations in identifying regional species, with low recall or precision for the Chinese Bamboo Partridge. This confirms that a large classifier trained based on large, publicly available datasets may struggle to generalize to regional species.

In our study, we achieved improved detection accuracy by using BirdNET detection results as input and retraining a random forest classifier based on features of the sound segments. This demonstrates that for regional species, a two-stage approach that combines large classifier results with localized, species-specific modeling can substantially enhance reliability.

Passive acoustic monitoring strengthens conservation through scalable, non-invasive data collection. Our analysis of vocal activity patterns illustrates how automated detection can generate continuous, spatially explicit data on species presence and diurnal and seasonal activities. This provides a foundation for evidence-based conservation, including population trend assessment, protected area management, and impact evaluation of environmental changes.

This study offers a practical framework for using BirdNET in regional species: Researchers should treat BirdNET outputs not as final detections, but as a prescreening layer for further validation. Combination with a regional dataset is essential for building a robust secondary classifier. At the same time, we should encourage and contribute regional recordings to public repositories to improve future model robustness.

## Figures and Tables

**Figure 4:**
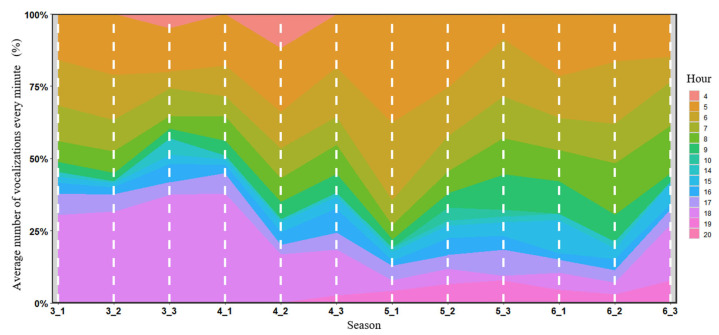
Diurnal pattern of vocal activity of the Chinese Bamboo Partridge in different seasons in a forest in eastern China. The diurnal patterns of vocal activity are expressed as the average number of sound segments detected per minute. Each group is represented by the hours’ relative percentage of vocalizations in days of different seasons. The longer the position occupied on the y-axis, the more likely the presence of the species in this hour is. Vocal activity was monitored via autonomous recording units from 1 March 2024 to 25 June 2024 at three acoustic monitoring stations. The percentage of vocalizations detected every hour can be found in Table S3. The data in each month were divided into three parts; for example, the data from 1 March to 10 March were marked as 3-1 season, and the data from 11 March to 20 March were marked as 3-2 season.

**Table 1:** Confusion matrix of the ability of 2 BirdNET models to correctly detect the presence of Chinese Bamboo Partridge in dataset 2. The test datasets were manually reviewed.

		BirdNET-Analyzer		BirdNET-Analyzer-XHS	
		Dataset 2		Dataset 2	
		Detected	Not Detected	Detected	Not Detected
human	presence	216 (TP)	1087 (FN)	1271 (TP)	32 (FN)
	absence	3 (FP)	X (TN)	1233 (FP)	X (TN)

**Table 2:** Confusion matrix of the ability of BirdNET and RF models to correctly detect the presence of Chinese Bamboo Partridge in dataset 3. The test datasets were manually reviewed.

		BirdNET-Analyzer-XHS		RF		BirdNET-Analyzer-XHS + RF	
		Dataset 3		Dataset 3 (Positive Predictions of BirdNET-Analyzer-XHS)		Dataset 3	
		Detected	Not Detected	Detected	Not Detected	Detected	Not Detected
human	presence	851 (TP)	30 (FN)	640 (TP)	211 (FN)	640 (TP)	241 (FN)
	absence	583 (FP)	X (TN)	220 (FP)	363 (TN)	220 (FP)	363 (TN)

## Data Availability

The data will be provided upon request to the authors.

## Supplementary Materials

The following supporting information can be downloaded at https://www.mdpi.com/article/10.3390/ani16020303/s1. Figure S1. Locations of the acoustic monitoring stations in Xiaohuangshan (Xinbei District, Changzhou City, Jiangsu Province, China). The inset shows the location of the study area (Xiaohuangshan) in Jiangsu Province, China; Figure S2. The recorder used in this study: the TN-Bt1 acoustic monitoring equipment (Tianning company, Jiangsu province, China). The recorder is waterproof (IP68 rating) and has a single internal omnidirectional microphone with a sensitivity of −26 dB. The signal-to-noise ratio (SNR) of the microphone is above 64 dB. It can set four schedules, with start time, end time, recording duration, sleep duration, and sampling rate. The highest sampling rate is 96 kHz. The diameter of the recorders can collect sound effectively within 50 m. The recording files were saved as WAV format in a Micro SD card; the biggest SD card storage capacity is 512 GB. With 15 new AA batteries, it can operate for over 300 h. The operating temperature is between −20 °C and 70 °C; Figure S3. Diurnal pattern of vocal activity of the Chinese Bamboo Partridge (*Bambusicola thoracicus*) in a forest in eastern China. The diurnal patterns of vocal activity are expressed as the average number of sound segments detected per minute. Vocal activity was monitored via autonomous recording units from 1 March 2024 to 25 June 2024 at three acoustic monitoring stations. The percentage of vocalizations detected every hour can be found in Table S3. The data in each month were divided into three parts; for example, the data from March 1st to March 10th were marked as 3-1 season, and the data from March 11th to March 20th were marked as 3-2 season; Table S1. The distribution of 196 days in different ten-day periods and sites. The data in each month were divided into three parts; for example, the data from March 1st to March 10th were marked as 3-1 season, and the data from March 11th to March 20th were marked as 3-2 season; Table S2. Average number of vocalizations of the Chinese Bamboo Partridge per minute in different hours. Percentage of vocalizations detected per hour with respect to the total number of vocalizations is also shown; Table S3. Average number of Chinese Bamboo Partridge vocalizations detected per minute in a forest in eastern China in different seasons. The percentage of vocalizations detected per hour with respect to the total number of vocalizations in different seasons is also shown; Table S4. Number of Chinese Bamboo Partridge vocalizations detected per ten days at three monitoring stations in a forest in eastern China. The total number and percentage of mean vocalizations detected per ten days every day with respect to the total number of vocalizations are also shown. Vocal activity was monitored by acoustic monitoring from 1 March 2024 to 25 June 2024 at three acoustic recording stations.

## Abbreviations

The following abbreviations are used in this manuscript:

XHS	Xiaohuangshan
RF	Random Forest
TP	True Positive
TN	True Negative
FP	False Positive
FN	False Negative
