# Supplementary material for: Patterns of Vocal Activity of the Chinese Bamboo Partridge Using BirdNET Analyzer
Source: Animals (Basel). 2026 Jan 19;16(2):303. doi: 10.3390/ani16020303 (PMC12838248; doi:10.3390/ani16020303)
Supplement: Supplementary file 1 [file animals-16-00303-s001.zip › animals-4062750-supplementary.pdf]

The Study of Diurnal and Seasonal Patterns of Vocal Activity of the Chinese Bamboo Partridge (*Bambusicola thoracicus*) in a Forest of Eastern China

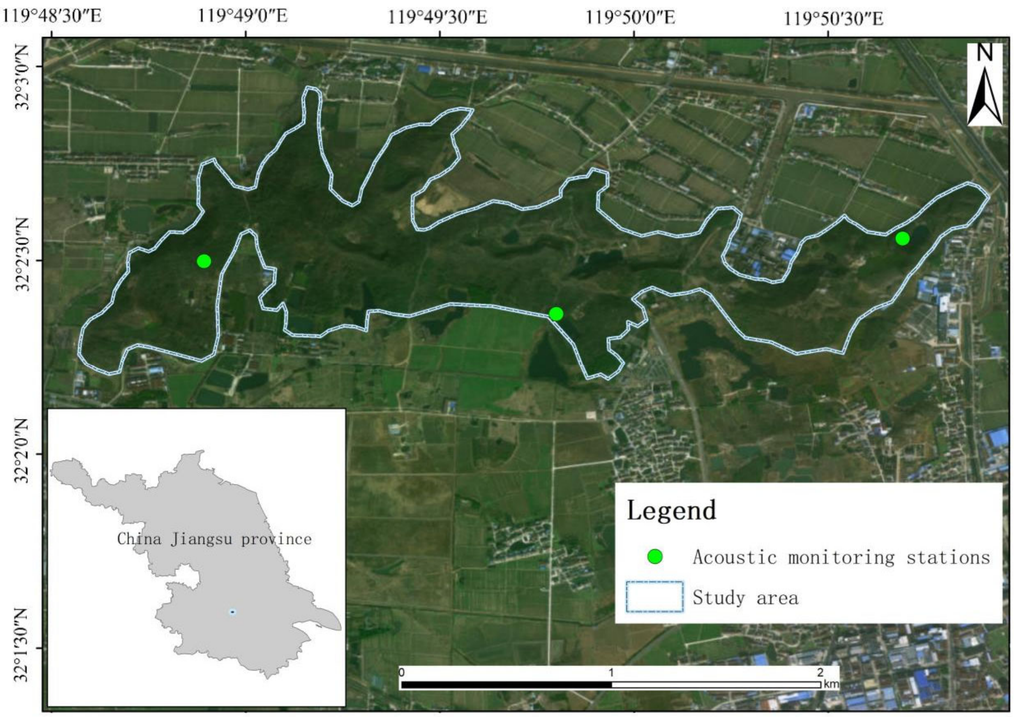

Figure S1 Locations of the acoustic monitoring stations in Xiaohuangshan (Xinbei District, Changzhou City, Jiangsu Province, China). The inset shows the location of the study area (Xiaohuangshan) in Jiangsu Province, China

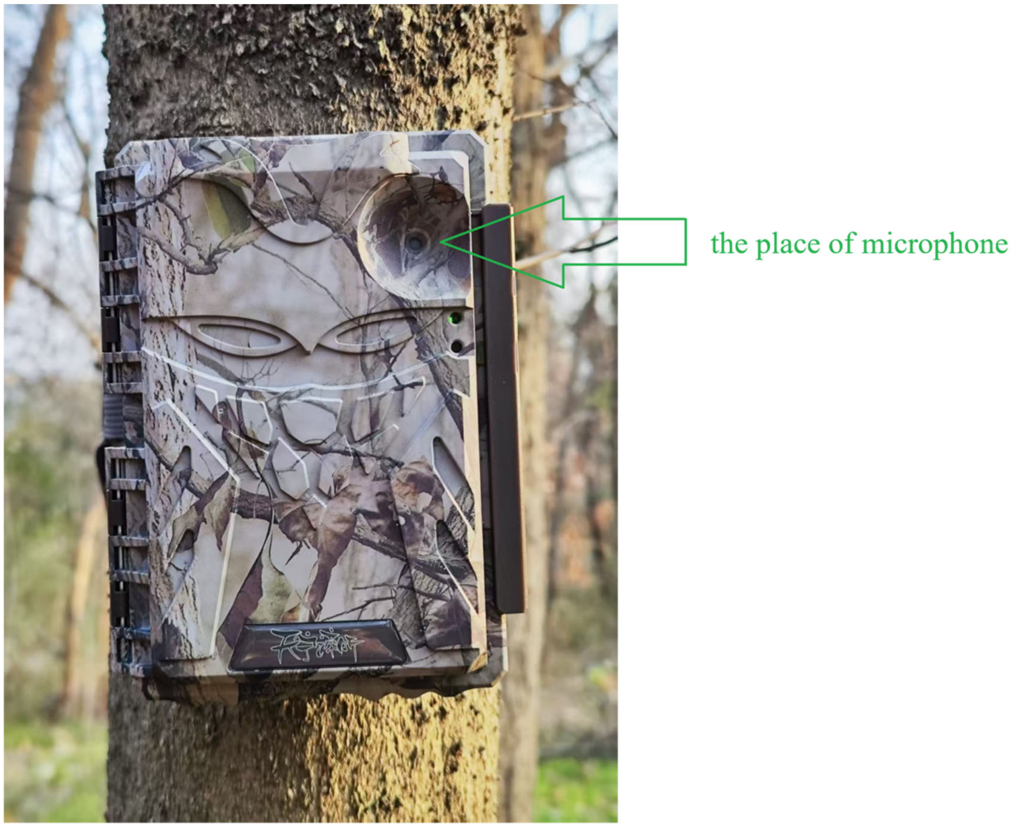

Figure S2 The recorder used in this study, the TN-Bt1 acoustic monitoring equipment (Tianning company, Jiangsu province, China). The recorder is waterproof (IP68 rating) and has a single internal omnidirectional microphone with a sensitivity of -26 dB.The signal-to-noise ratio (SNR) of microphone is above 64dB. It can set four schedules, with start time, end time, recording duration, sleep duration and sampling rate. The highest sampling rate is 96 kHz. The diameter of the recorders can collect sound effectively within 50 meters. The recording files were saved as WAV format in Micro SD card, the biggest SD card storage capacity is 512GB. With 15 new AA batteries, it can operate over 300 hours. The operating temperature is between -20℃ and 70℃.

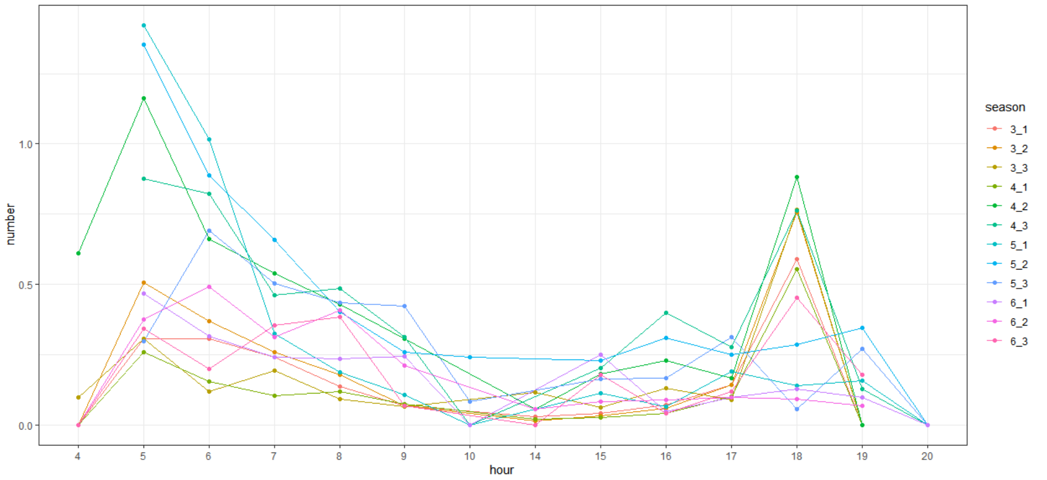

Figure S3 Diurnal pattern of vocal activity of the Chinese Bamboo-Partridge (*Bambusicola thoracicus*) in a forest of eastern China. The diurnal patterns of vocal activity are expressed as the average number of sound segments detected per minute. Vocal activity was monitored via autonomous recording units from 1 March 2024 to 25 June 2024 at three acoustic monitoring stations. The percentage of vocalizations detected every hour can be found in Table S3. The data in each month were divided into three parts, for example, the data from March 1st to March 10th were marked as 3-1 season, and the data from March 11th to March 20th were marked as 3-2 season.

Table S1 The distribution of 196 days in different ten-days and site. The data in each month were divided into three parts, for example, the data from March 1st to March 10th were marked as 3-1 season, and the data from March 11th to March 20th were marked as 3-2 season.

Season	XHS05_Days	XHS06_Days	XHS10_Days	Total_Days
3_1	9	8	3	20
3_2	5	7	10	22
3_3	4	4	2	10
4_1	10	9	1	20
4_2	7	7	10	24
4_3	4	2	4	10
5_1	3	1	0	4
5_2	10	10	9	29
5_3	2	4	6	12
6_1	6	7	1	14
6_2	9	8	10	27
6_3	0	0	4	4
TOTAL	69	67	60	196

Table S2 Average number of vocalizations of Chinese Bamboo Partridge per minutes in different hours. Percentage of vocalizations detected per hour with respect to the total number of vocalizations are also shown.

Hour	average number of vocalizations per minutes	%
4:00	0.10	2.98%
5:00	0.66	19.75%
6:00	0.51	15.22%
7:00	0.37	11.02%
8:00	0.30	9.00%
9:00	0.19	5.82%
10:00	0.16	4.81%
14:00	0.04	1.25%
15:00	0.13	3.79%
16:00	0.15	4.44%
17:00	0.16	4.92%
18:00	0.47	14.06%
19:00	0.10	2.94%
20:00	0.00	0.00%
TOTAL	3.33	

Table S3 Average number of Chinese Bamboo Partridge vocalizations detected per minute in a forest of eastern China in different seasons. The percentage of vocalizations detected per hour with respect to the total number of vocalizations in different seasons are also shown.

Season	Hour	average number of vocalizations per minutes	%
3_1	4:00	0.000	0.0%
3_1	5:00	0.306	15.8%
3_1	6:00	0.306	15.8%
3_1	7:00	0.241	12.4%
3_1	8:00	0.138	7.1%
3_1	9:00	0.070	3.6%
3_1	14:00	0.029	1.5%
3_1	15:00	0.042	2.2%
3_1	16:00	0.071	3.7%
3_1	17:00	0.144	7.4%
3_1	18:00	0.590	30.5%
3_1	19:00	0.000	0.0%
3_2	4:00	0.000	0.0%
3_2	5:00	0.506	21.1%
3_2	6:00	0.370	15.5%
3_2	7:00	0.260	10.9%
3_2	8:00	0.179	7.5%
3_2	9:00	0.068	2.8%
3_2	14:00	0.017	0.7%
3_2	15:00	0.033	1.4%
3_2	16:00	0.059	2.5%
3_2	17:00	0.145	6.0%
3_2	18:00	0.757	31.6%
3_2	19:00	0.000	0.0%
3_3	4:00	0.100	4.9%
3_3	5:00	0.308	15.1%
3_3	6:00	0.118	5.8%
3_3	7:00	0.195	9.5%
3_3	8:00	0.092	4.5%
3_3	9:00	0.067	3.3%
3_3	14:00	0.117	5.7%
3_3	15:00	0.063	3.1%
3_3	16:00	0.130	6.4%
3_3	17:00	0.090	4.4%
3_3	18:00	0.767	37.5%
3_3	19:00	0.000	0.0%
4_1	4:00	0.000	0.0%
4_1	5:00	0.260	17.8%
4_1	6:00	0.154	10.6%
4_1	7:00	0.103	7.1%
4_1	8:00	0.121	8.3%
4_1	9:00	0.074	5.1%
4_1	14:00	0.020	1.4%

4_1	15:00	0.028	1.9%
4_1	16:00	0.043	3.0%
4_1	17:00	0.101	6.9%
4_1	18:00	0.553	38.0%
4_1	19:00	0.000	0.0%
4_2	4:00	0.611	11.7%
4_2	5:00	1.162	22.2%
4_2	6:00	0.660	12.6%
4_2	7:00	0.539	10.3%
4_2	8:00	0.429	8.2%
4_2	9:00	0.307	5.9%
4_2	14:00	0.056	1.1%
4_2	15:00	0.183	3.5%
4_2	16:00	0.228	4.4%
4_2	17:00	0.167	3.2%
4_2	18:00	0.883	16.9%
4_2	19:00	0.000	0.0%
4_3	4:00	0.000	0.0%
4_3	5:00	0.877	18.6%
4_3	6:00	0.822	17.4%
4_3	7:00	0.463	9.8%
4_3	8:00	0.485	10.3%
4_3	9:00	0.312	6.6%
4_3	10:00	0.000	0.0%
4_3	15:00	0.202	4.3%
4_3	16:00	0.398	8.4%
4_3	17:00	0.277	5.9%
4_3	18:00	0.762	16.1%
4_3	19:00	0.128	2.7%
4_3	20:00	0.000	0.0%
5_1	4:00	0.000	0.0%
5_1	5:00	1.422	38.1%
5_1	6:00	1.017	27.3%
5_1	7:00	0.325	8.7%
5_1	8:00	0.188	5.0%
5_1	9:00	0.108	2.9%
5_1	10:00	0.000	0.0%
5_1	15:00	0.112	3.0%
5_1	16:00	0.067	1.8%
5_1	17:00	0.192	5.1%
5_1	18:00	0.142	3.8%
5_1	19:00	0.158	4.2%
5_1	20:00	0.000	0.0%
5_2	4:00	0.000	0.0%
5_2	5:00	1.352	25.9%
5_2	6:00	0.888	17.0%
5_2	7:00	0.658	12.6%
5_2	8:00	0.402	7.7%

5_2	9:00	0.259	5.0%
5_2	10:00	0.241	4.6%
5_2	15:00	0.229	4.4%
5_2	16:00	0.311	6.0%
5_2	17:00	0.251	4.8%
5_2	18:00	0.287	5.5%
5_2	19:00	0.345	6.6%
5_2	20:00	0.000	0.0%
5_3	4:00	0.000	0.0%
5_3	5:00	0.297	8.7%
5_3	6:00	0.690	20.3%
5_3	7:00	0.503	14.8%
5_3	8:00	0.436	12.8%
5_3	9:00	0.422	12.4%
5_3	10:00	0.083	2.4%
5_3	15:00	0.165	4.9%
5_3	16:00	0.168	4.9%
5_3	17:00	0.313	9.2%
5_3	18:00	0.056	1.6%
5_3	19:00	0.271	8.0%
5_3	20:00	0.000	0.0%
6_1	4:00	0.000	0.0%
6_1	5:00	0.468	21.9%
6_1	6:00	0.315	14.8%
6_1	7:00	0.243	11.4%
6_1	8:00	0.235	11.0%
6_1	9:00	0.244	11.5%
6_1	10:00	0.000	0.0%
6_1	15:00	0.250	11.7%
6_1	16:00	0.049	2.3%
6_1	17:00	0.099	4.6%
6_1	18:00	0.129	6.0%
6_1	19:00	0.100	4.7%
6_1	20:00	0.000	0.0%
6_2	4:00	0.000	0.0%
6_2	5:00	0.376	16.4%
6_2	6:00	0.492	21.5%
6_2	7:00	0.314	13.7%
6_2	8:00	0.407	17.8%
6_2	9:00	0.213	9.3%
6_2	14:00	0.056	2.4%
6_2	15:00	0.083	3.6%
6_2	16:00	0.090	3.9%
6_2	17:00	0.098	4.3%
6_2	18:00	0.092	4.0%
6_2	19:00	0.068	3.0%
6_3	4:00	0.000	0.0%
6_3	5:00	0.342	14.7%

6_3	6:00	0.200	8.6%
6_3	7:00	0.354	15.2%
6_3	8:00	0.383	16.5%
6_3	9:00	0.070	3.0%
6_3	14:00	0.000	0.0%
6_3	15:00	0.179	7.7%
6_3	16:00	0.042	1.8%
6_3	17:00	0.121	5.2%
6_3	18:00	0.454	19.5%
6_3	19:00	0.180	7.7%

Table S4 Number of Chinese Bamboo Partridge vocalizations detected per ten-days at three monitoring stations in a forest of eastern China. The total number and percentage of mean vocalizations detected per ten-days everyday with respect to the total number of vocalizations are also shown. Vocal activity was monitored by acoustic monitoring from 1 March 2024 to 25 June 2024 at three acoustic recording stations.

Season	XHS05	XHS06	XHS10	Total	Total_Days	Average	%
3_1	1100	678	500	2278	20	113.9	5.4%
3_2	572	639	1914	3125	22	142.0	6.7%
3_3	298	315	496	1109	10	110.9	5.2%
4_1	880	545	297	1722	20	86.1	4.1%
4_2	1741	766	4059	6566	24	273.6	12.9%
4_3	789	259	1785	2833	10	283.3	13.3%
5_1	562	321	0	883	4	220.8	10.4%
5_2	3099	2953	2509	8561	29	295.2	13.9%
5_3	421	871	1090	2382	12	198.5	9.3%
6_1	721	758	310	1789	14	127.8	6.0%
6_2	644	722	2242	3608	27	133.6	6.3%
6_3	0	0	555	555	4	138.8	6.5%
TOTAL	10827	8827	15757	35411	196	2124.5	
